# Multilevel factor analysis of flipped classroom in dental education: A 3-year randomized controlled trial

**DOI:** 10.1371/journal.pone.0257208

**Published:** 2021-09-10

**Authors:** Zuo Wang, Eiko Yoshida Kohno, Kenji Fueki, Takeshi Ueno, Yuka Inamochi, Kazuki Takada, Noriyuki Wakabayashi

**Affiliations:** 1 Department of Removable Partial Prosthodontics, Graduate School, Tokyo Medical and Dental University (TMDU), Tokyo, Japan; 2 Department of Professional Development in Health Sciences, Graduate School of Medical and Dental Sciences, Tokyo Medical and Dental University (TMDU), Tokyo, Japan; University of South Australia, AUSTRALIA

## Abstract

**Purpose:**

Previous studies have rarely attempted to test the confounding factors that may affect learning outcomes of the flipped classroom. The purpose of this study was to assess how flipped classrooms affect the acquisition of knowledge in clinical dental education based on multilevel factor analysis.

**Method:**

The authors conducted a 3-year (2017, 2018, and 2019) randomized controlled trial in a series of introductory prosthodontics courses in dental education. A total of 137 participants were randomly assigned to flipped classroom (n = 70, 51%) or lecture (n = 67, 49%) formats. The flipped group was instructed to self-learn knowledge-based content through online preparation materials, including videos and text, while the lecture group was given text only. Both groups were provided with the same study content and opportunities for different styles of learning. The session attendance rate and number of times the materials were accessed were monitored. Individual and team readiness assurance tests (IRAT/TRAT) were conducted to evaluate knowledge acquisition. A multilevel linear regression analysis was conducted on both instructional styles (flipped vs. lecture) as an intervention factor, and confounding factors that could affect the outcomes were implemented.

**Results:**

The average number of online accesses was 2.5 times per session in the flipped group and 1.2 in the lecture group, with a significant difference (p < .05). The average IRAT score was significantly higher in the flipped than in the lecture group (effect size [ES] 0.58, p < .001). The number of online accesses was significantly and positively correlated with IRAT scores (0.6 [0.4, 0.8]). The instructional style was significantly and positively correlated with TRAT scores (coefficient [95% confidence interval]: 4.6 [2.0, 7.3]), but it was not correlated with IRAT (4.3 [-0.45, 9.0]).

**Conclusions:**

The flipped classroom was more effective than the lecture format regarding knowledge acquisition; however, the decisive factor was not the instructional style but the number of individual learning occasions. The employment of the flipped classroom was the decisive factor for team-based learning outcomes.

## Introduction

The term “flipped classroom” refers to an instructional style that has rapidly become popular in the context of health care education [1]. Prior to classroom sessions, students independently learn foundational content through homework assignments to acquire lower-level learning objectives such as *fact remembering*. This is often accomplished by videos and other electronic media. However, in class, students engage in instructor-facilitated learner-centered activities to obtain higher cognitive abilities, such as *applying* and *analyzing* [2–4]. The spread of this instructional style has not been hindered by the COVID-19 pandemic; on the contrary, it has been even spreading by the need for distance learning and the educators who are willing to provide students with an active learning opportunity to apply their knowledge [5]. However, before we substantially transform the delivery of undergraduate health care education, the effectiveness of the flipped classroom needs to be fully assessed by comparing it with the conventional didactic lecture format.

Studies have produced conflicting results regarding the effect of flipped classrooms on student performance. While reports from the dental education field have shown overall positive effects [6, 7], research has also indicated that there are no significant differences between the flipped classroom and lecture format based on student test scores for case-based questions [8]. This disparity in results is partly due to the varied assessment outcomes used between studies. Specific outcome measures used in the field of medical education include multiple-choice tests [9, 10], feedback comments [11], clinical examinations [12], final grades [13], and both multiple-choice and clinical-skill tests [14, 15]. Generally, studies only implement one outcome, which makes it difficult to compare their results. More importantly, we also speculate that the effectiveness of the flipped classroom is not fully understood because, to the best of our knowledge, no previous studies have attempted to test the confounding factors that may affect learning outcomes. The review papers summarized outcomes of the flipped classroom strategy in various study environments of previous observational and comparative studies [16, 17], however; the publications used in those meta-analyses rarely tested the effect of confounding factors that might potentially affect the learning outcomes of the flipped strategy.

The benefit of this instructional style is supposedly based on active learning [18], which is often represented by a positive influence on the emotional factors associated with the interactive sessions [10]. However, since the flipped classroom requires students to do homework, they tend to spend more time learning, which could be the real reason they tend to gain higher scores on assessments [19]. Moreover, relatively little research has been conducted to assess the factors that influence the effectiveness of the flipped classroom. Such analysis should be based on the principles of multivariate statistics, which involve the observation and analysis of more than one statistical outcome variable at a time.

This 3-year randomized controlled trial evaluated how the flipped classroom affected the acquisition of knowledge based on multilevel factor analysis, specifically through a comparison with the lecture format in the context of an introductory prosthodontics course in clinical dental education. This enabled us to identify the dominant factors that influenced the learning outcomes.

## Methods

### Participant enrollment

A single-center, year-stratified, two-arm, single-assessor-blinded trial was planned. The eligibility criteria were as follows: students in the fourth grade of a 6-year Doctor of Dental Surgery (DDS) program at a dental school, specifically for the fiscal years 2017, 2018, and 2019. The trial began with the recruitment of 1st-year participants in October 2017 and ended with the term-end examination of last-year participants in February 2020. Upon recruitment, all students received verbal and written information about the trial from the research coordinator (E.Y.K.), independent of teaching. Each student provided written informed consent prior to participation. They were then asked to report any previous experience with the flipped classroom format [20] and rate their level of interest [21] in removable prosthodontics, based on the following question: “Do you agree with the statement that you are looking forward to studying removable prosthodontics?” Answers were submitted according to a multiple-choice scale (strongly agree, agree, neutral, disagree, and strongly disagree). Prior to study commencement, the protocol was approved by the Institutional Review Board of the Tokyo Medical and Dental University (TMDU) (approval no. D2017-024) and registered in the UMIN Clinical Trials Registry (www.umin.ac.jp/) (UMIN000028111, registered in 01/ 09/ 2017). This study was conducted in accordance with the Declaration of Helsinki.

### Randomization and allocation

Participants were allocated to one of two instructional groups (flipped or lecture) via simple randomization. Specifically, a computerized random number ranging from 0 to 1 was generated for each participant. Those who were assigned numbers <0.5 were allocated to the lecture group, while those with numbers ≥0.5 were allocated to the flipped group. These number generations and allocations were conducted by the research coordinator, who concealed the results from the teachers. Participants who objected to their designated group were allowed to switch groups, but their data were analyzed as part of the originally designated group (intention-to-treat analysis).

### Blinding

This was a single-blinded trial, meaning that researchers who assessed the outcomes and analyzed the data were blinded to the allocation, whereas participants were aware of their allocation.

### Sample size

Based on a review report detailing differences in the learning effects between the lecture format and active-learning approach (e.g., problem-based learning and group problem-solving; no report on flipped classroom) [22], student performance on examinations and concept inventories increased on average by 0.5 of the effect size under active learning. Thus, the sample size was calculated at 128 participants (64 per group), with a two-tailed 5% significance level and a power of 80% (G*Power). A total of three consecutive academic years were required to recruit this number of participants.

### Interventions

**Fig 1:**
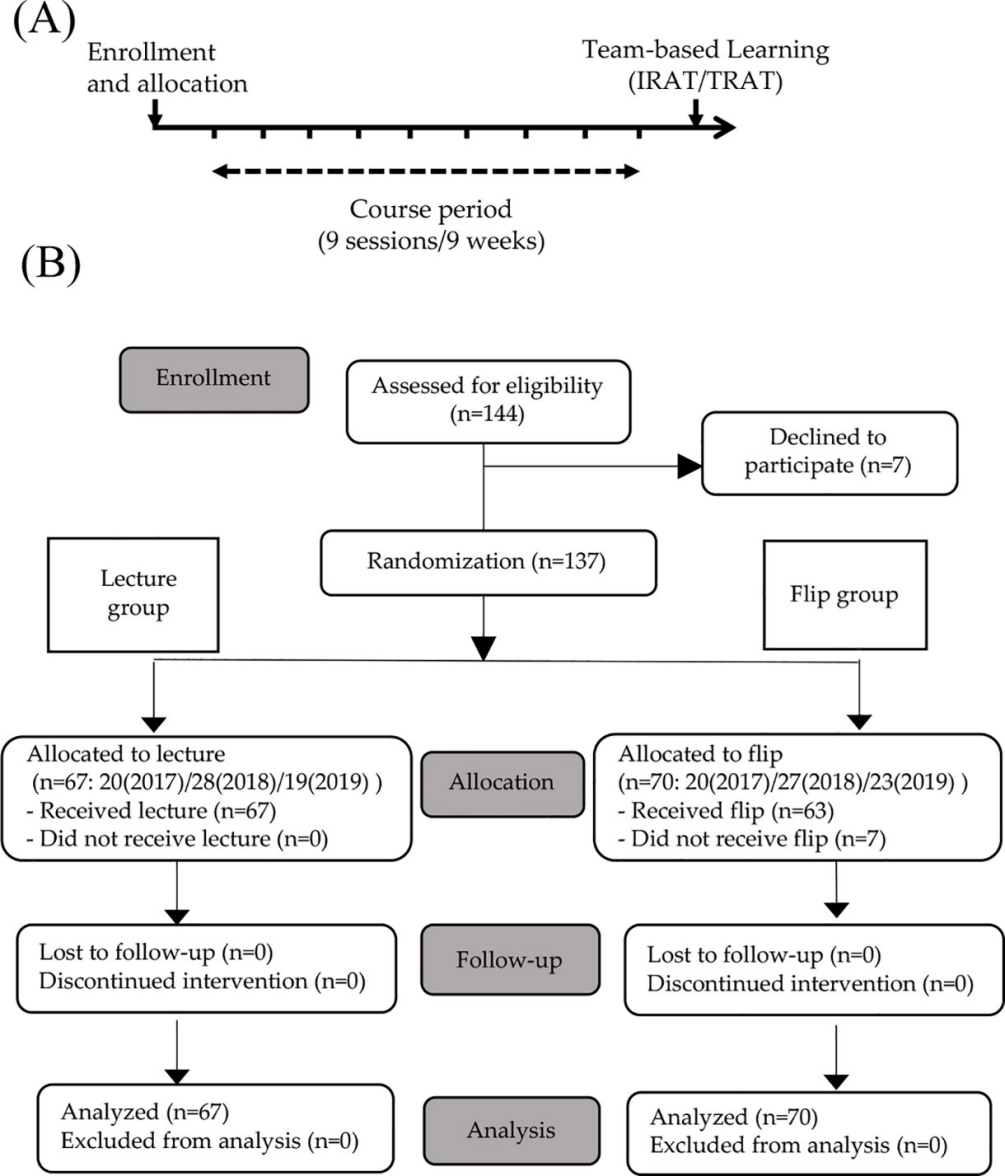
Timeline and participants flow. (A) Timeline of this study. IRAT, individual readiness assurance test; TRAT, team readiness assurance test. (B) Participants flow until analysis.

The introductory removable prosthodontics course included nine 3-hour sessions in nine weeks, beginning in October and ending in early December in 2017, 2018, and 2019 (Fig 1). Each participant attended the assigned sessions throughout the course period. Each week, two simultaneous sessions (flipped and lecture) were conducted independently in separate rooms. One of the three experienced teachers (N.W., K.F., and T.U.) was randomly assigned to either of the two sessions each week. The academic content of all nine sessions was identical for both groups. Because learning effects may vary based on academic content [23], this study was implemented through a series of sessions covering broad areas of clinical dentistry, including oral anatomy, epidemiology, oral mucosal diseases, prosthodontic materials, occlusion and masticatory functions, treatment planning, denture design, clinical and laboratory procedures, and post-treatment maintenance.

### Flipped teaching sessions

Participants were able to stream and/or download relevant text documents and videos, which were uploaded to the university’s e-learning system from one week prior to each session until the end of the term. Each video was provided in mpeg4 format, with a 40-minute-long presentation based on PowerPoint slides narrated by one teacher (N.W.). Each text document covered a summary of the contents of each session, provided in.pdf format. Participants were instructed to self-study using text documents and videos prior to each session. During the session, they worked on eight to ten multiple-choice quizzes in small groups [24]. The quizzes were mixtures of knowledge- and clinical case-based scenarios, with all answers and relevant facts having been presented through the videos and texts. On a voluntary basis, a few participants were asked to give mini lectures on important topics for each session. However, the teacher did not give lectures, instead served as a facilitator, provided summaries of the quiz answers, and occasionally presented relevant clinical cases involving actual patients via intraoral records and photographs.

### Lecture sessions

The lecture group used the same text documents as the flipped group, but they were not provided lecture videos throughout the semester. Participants could access and download the text documents in pdf format from one week prior to each session until the end of the term. Each 3-hour lecture was given with the same projected slides used in the narrated videos presented to the flipped group so that the same study contents were provided to both groups. Participants were occasionally presented with relevant clinical cases involving actual patients. During the lecture, the participants were not given quizzes, but were provided with the same study contents included in them. No interactive communication was evoked during the lectures, except when dealing with voluntary questions from the participants. The lecturers of the two simultaneous sessions carefully confirmed that the two groups received the same study contents by checking the slides, text, and quiz regime prior to each session.

### Team-based learning

Participants in both groups attended the same team-based learning (TBL) session one week after the final flipped and lecture sessions. The teams were arranged so that participants from the two groups did not mix directly. Each participant was given 40 minutes to answer 27 multiple-choice questions independently in a closed-book manner (individual readiness assurance test: IRAT). The test was given to individual students to evaluate lower- to higher-order cognitive skills based on Bloom’s taxonomy of cognitive domains [4]. The questions covered a broad area from basic knowledge of terminology to diagnosis, treatment options, and clinical/laboratory procedures through case scenarios. The questions were selected from a stock of reserve questions that were clearly distinguished from the quizzes used in the flipped sessions. The coordinator carefully checked the questions prior to TBL so that knowing the answer to the quizzes in the flipped sessions would not favor the answer to the IRAT.

Participants were then divided into pre-assigned teams containing 4–6 students to work on the same questions presented in the IRAT for 60 minutes, but in an open-book manner. Each team submitted only one answer sheet (team readiness assurance test: TRAT). The TRAT was followed by a review of all questions, which was conducted by one of the teachers, randomly selected. The TBL in this study was conducted to review the academic content of all sessions. There were no subsequent application or appeal sessions, which generally followed the IRAT/TRAT [25].

All answers were scored by a research coordinator. All questions were renewed every year. The outcome measures (IRAT and TRAT as the primary and secondary outcomes, respectively) were converted to scores ranging from 0 to 100 based on the percentages of correct answers.

### Student behaviors

For each participant, the session attendance rate (SAR) was calculated as the number of attended hours divided by the total course hours [9]. Each online access (log-in) of the preparation materials (video and text for the flip, text for the lecture group) located in the e-learning system was counted as a learning occasion during the course period. However, we could not trace learning that was accomplished via downloaded text documents and/or videos on PCs/tablets. Therefore, these instances were not considered.

### Statistical method

Bivariate analyses were conducted to compare the learning activities and performance of the outcome measures between the two groups. We then conducted multilevel linear regression (multivariate) analyses to reveal the effects of each instructional style (flip/lecture, a fixed effect) on IRAT and TRAT. The number of online accesses and SAR were set as confounding factors with fixed effects, while the academic year was set as a multilevel variance with random effects. Stata 15 (Stata Corp LP, College Station, TX, USA) was used for all statistical analyses. Statistical significance was set at p < 0.05.

## Results

### Randomization and study participation

Over the 3-year study period, a total of 137 students agreed to participate, while seven (4.9%, 7/144) declined and were therefore not randomized (Fig 1). As mentioned earlier, participants were randomly allocated to the flipped (n = 70, 51%) or lecture (n = 67, 49%) groups. Following this procedure, seven participants in the flipped group (10%, 7/70) objected to their allocations and were thus switched to the lecture group, but were still analyzed as part of the flipped group. None of the participants dropped the course or submitted requests to change groups during the course period. Regarding the pre-study questions, seven participants each in the flipped (10%, 7/70) and lecture (10%, 7/67) groups had previous experience with flipped classrooms. The level of interest in removable prosthodontics were also similar in both groups; 75% of the flipped and 77% of the lecture group participants answered “strongly agree” or “agree” to the question using a multiple-choice scale. As other baseline characteristics such as gender and previous grade repetition were similar between groups, these data are not shown.

### Participant behaviors

**Fig 2:**
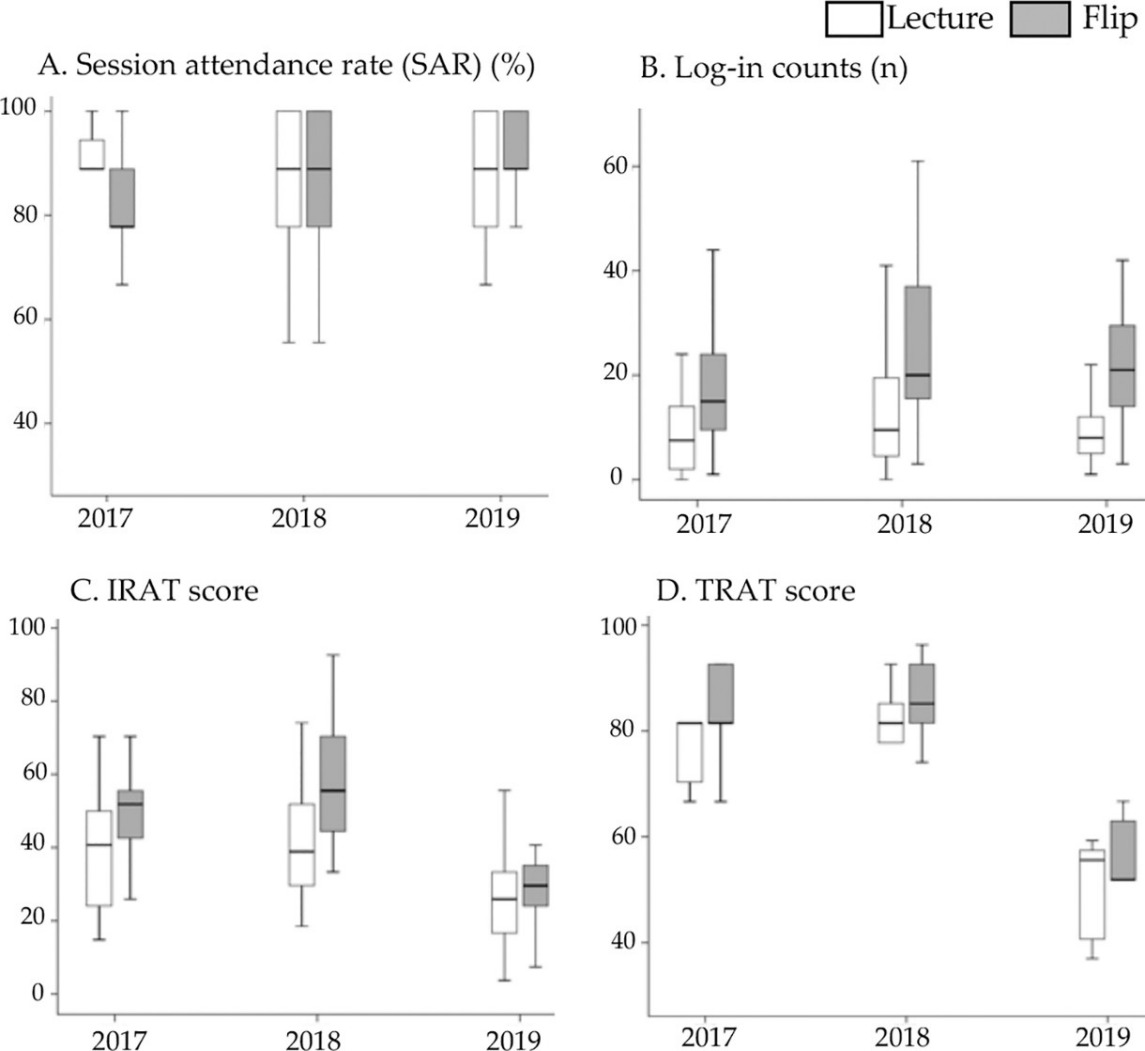
Participant behaviors and learning outcomes. Boxplots of participant behaviors (A, B) and learning outcomes (C, D). A, session attendance rate (%); B, number of online accesses (log-in counts) during the course period; C, IRAT (individual readiness assurance test) score; D, TRAT (team readiness assurance test) score.

There were no significant differences in SAR between groups (Fig 2), except for the year 2017, when the flipped group showed significantly smaller SAR than the lecture group (p < .05). Statistical significance was not shown for individual years. During the course period, the average number of online accesses was 2.5 times per session in the flipped group and 1.2 in the lecture group, with a significant difference (p < .05) (Table 1).

**Table 1:** Participant behaviors and outcome scores.

Behaviors/Scores	Mean (SD) Scores		Effect Size	P-value
	Lecture (n = 67)	Flipped (n = 70)		
Session attendance rate (SAR) (%)	85 (16)	86 (13)	/	0.703
Number of online accesses (log-in counts) during the course period	11.1 (9.9)	22.2 (13.1)	/	<0.001
IRAT(individual readiness assurance test)	36.1 (15.8)	46.1 (18.3)	0.58	0.001
TRAT (team readiness assurance test)	71.6 (15.7)	76.0 (15.3)	0.28	0.102

An independent t-test was used to examine IRAT and TRAT scores between the lecture and flipped groups.

### Test scores

The average IRAT score was significantly higher (t-test) in the flipped than in the lecture group (Table 1, effect size [ES] 0.58, p < .001). The average rate of increase from IRAT to TRAT scores was 98% and 65% for the lecture and the flipped group, respectively (Fig 2). The average TRAT score was significantly higher (t-test) in the flipped than in the lecture group, while the difference between the two groups was not statistically significant in the total 3 years.

### Multivariate analyses

The instructional style was significantly and positively correlated with TRAT scores (coefficient [95% confidence interval]: 4.6 [2.0, 7.3]) when adjusted for the number of online accesses and SAR, but it was not correlated with IRAT (4.3 [-0.45, 9.0]) (Table 2). Furthermore, the number of online accesses was significantly and positively correlated with the IRAT scores (0.6 [0.4, 0.8]). As a random effect, academic year was significantly correlated with all outcomes (p < .001).

**Table 2:** Multilevel linear regression analysis.

Test	Fixed effects	Coefficient (95% CI)		Effect Size	P-value
IRAT^#^	Instructional type (ref: Lecture)	4.25	(-0.45, 8.96)	0.15	0.076
	Number of online accesses (log-in counts)	0.59	(0.40, 0.79)	0.50	<0.001
	Session attendance rate (%)	0.02	(-0.14, 0.17)	0.02	0.849
	Constant	27.27	(10.52, 44.03)	/	0.001
TRAT^#^	Instructional type (ref: Lecture)	4.61	(1.96, 7.27)	0.29	0.001
	Number of online accesses (log-in counts)	0.10	(-0.01, 0.21)	0.15	0.084
	Session attendance rate (%)	0.04	(-0.05, 0.13)	0.08	0.351
	Constant	65.17	(48.00, 82.35)	/	<0.001

CI = confidence interval

# Random effect: Academic year (p < 0.001)

## Discussion

The flipped classroom was significantly more effective for acquisition of knowledge than the lecture format, as indicated by the IRAT score (Table 1). Multivariate analysis indicated that the number of online accesses was significantly and positively correlated with IRAT scores, while the instructional style was not (Table 2). The results suggested that the flipped classroom style of instruction did not improve knowledge acquisition by itself. Previous studies confirmed that the requirement to watch lectures followed by an hour-long discussion essentially put students in flipped class in significantly more time than those in lecture courses, which might have contributed to positive effects on overall performances [9, 26]. In those studies, however, the influence of the factor on the outcome was not tested statistically. The present study employed potential confounding factors including the number of online accesses/occasions to judge if these factors had significant influence on the learning outcome. The findings indicated that participants in the flipped group accessed the preparation materials more frequently than reported in a previous study [27] and engaged in more individual learning than the lecture group. The correlation between increased study opportunities and higher IRAT scores concur with previous research showing that homework is a critical factor for increasing knowledge-based performance [9]. Other studies have suggested that the flipped classroom is advantageous because its instructional style allows students to study at their own pace, while using digital educational materials [20, 28]. The present study found that the flipped classroom provided dental students with opportunities to develop their self-directed learning skills while enhancing their ability to consolidate acquired knowledge during in-class sessions.

While the flipped group had significantly higher IRAT scores than the lecture group, TRAT scores were comparable between the groups (Table 1). This was because there was a more pronounced score increase moving from IRAT to TRAT in the lecture group. The result suggests that productive collaboration might occur in TBL regardless of the instructional style, but the score increase was especially noticeable for the lecture group because of their lower individual scores at baseline. These results were closely related to a lower coefficient of variation (CV value: standard deviation/mean score) in IRAT scores for the flipped group (27% to 35%, depending on the year) when compared to the lecture group (36% to 50%), suggesting that collaborative learning reduces the range of score distributions [8]. Conversely, the multivariate analysis indicated that the instructional style was positively correlated with TRAT scores (Table 2). It should be noted that multivariate analysis was not applied to assess the effect of the team-based session in the TBL, but that of the instructional style. Therefore, it was not surprising that the average TRAT score of the flipped group was still higher than that of the lecture (without statistical significance). The results of multivariate analysis clearly showed that the dominant factor that made the difference between the two groups in the TRAT score was the instructional style, and that the flipped style of instruction was more effective. This result may be attributable to the higher overall knowledge background (i.e., IRAT scores) found in the flipped group prior to the team sessions in TBL and/or their experiences of collaborative sessions in the flipped classroom, which may have been cultivated through collaborative learning activities during flipped teaching sessions [2, 29]. The multivariate analysis revealed its most influential factor, that is, the flipped classroom style of instruction. This implication could not have been unveiled by means of comparative statistics between the flipped and lecture groups.

Multivariate analysis also showed that academic year was significantly correlated with all learning outcomes as a random effect (Table 2). Specifically, the findings in 2019 were quite different from other years in that it was the only year in which IRAT scores were not significantly different between groups, while TRAT scores were significantly different (not shown for individual years). Another notable finding was that the IRAT scores were very low for both groups in 2019 when compared to other years (Fig 2). Since the 2019 participants showed similar levels of interest in removable prosthodontics, the number of online accesses, and SAR when compared to participants from other years, we speculate that the questions were generally more challenging in 2019. Nevertheless, the flipped group still achieved significantly higher TRAT scores in 2019 than the lecture group. This may be attributable to their higher overall collaborative skills [30], which is consistent with earlier results showing that the flipped classroom significantly influenced performance during the team sessions.

Multivariate analysis indicated that the decisive factor for IRAT and TRAT scores was not the SAR during the class period (Table 2). The result seems to indicate that the acquisition of knowledge was achieved through self-learning without attending the actual classroom. However, this may not be true, especially for higher-order cognitive skills. Moreover, the achievements in clinical reasoning skills were positively correlated with the use of a flipped classroom setting in a previous study [31], in which instructors continually asked questions that required students to engage in higher-order thinking.

From the start of the course period, the flipped group students accessed the preparation files, attended the class, and answer questions without complaining of any difficulty in the learning method. On the other hand, students’ improvement of utilizing this instructional method was not found in the IRAT scores by subjects (earlier or later subjects). Therefore, the potential improvements of the learners’ skills was unlikely to have effects on the scores.

The present study’s findings should be interpreted carefully, as learning contents were somewhat limited during the flipped and lecture group sessions. Although, to address various knowledge backgrounds, the teachers implemented relevant clinical case-based scenarios during the in-class sessions [32], learning outcomes were not focused on the acquisition or sophistication of clinical reasoning skills; rather, emphasis was placed on the purpose of the introductory course. Therefore, it is not reasonable to conclude that the flipped classroom was not effective in helping students acquire higher-order cognitive skills. As an instructional style, the effectiveness of the flipped classroom requires further investigation under the umbrella of diverse active learning schemes [18, 33]. This includes the need for case studies during sessions. Nevertheless, our results provide new insight into the learning mechanism involved in flipped classrooms, while showing some of its limitations. Since it was clearly indicated in this study that the learning effect of the flipped classroom was closely related to the learning effort/occasions of each participant, it has become necessary to further understand the learning efficiency of this instructional mechanism. Our findings support the call for learning activities designed to improve higher-order cognitive skills in healthcare education.

## Supporting information

S1 Checklist CONSORT 2010 checklist of information to include when reporting a randomised trial. (DOC)
